# Addressing gross motor function by functional repetitive neuromuscular magnetic stimulation targeting to the gluteal muscles in children with bilateral spastic cerebral palsy: benefits of functional repetitive neuromuscular magnetic stimulation targeting the gluteal muscles

**DOI:** 10.3389/fneur.2023.1161532

**Published:** 2023-07-26

**Authors:** Leonie Grosse, Malina A. Späh, Corinna Börner, Julian F. Schnabel, Anne C. Meuche, Barbara Parzefall, Ute Breuer, Birgit Warken, Alexandra Sitzberger, Matthias Hösl, Florian Heinen, Steffen Berweck, Sebastian A. Schröder, Michaela V. Bonfert

**Affiliations:** ^1^LMU Hospital, Department of Pediatrics – Dr. von Hauner Children’s Hospital, Division of Pediatric Neurology and Developmental Medicine, Ludwig-Maximilians-Universität München, Munich, Germany; ^2^LMU Center for Children with Medical Complexity – iSPZ Hauner, Ludwig-Maximilians-Universität München, Munich, Germany; ^3^Gait and Motion Analysis Laboratory, Schoen Clinic Vogtareuth, Vogtareuth, Germany; ^4^Specialist Center for Pediatric Neurology, Neurorehabilitation and Epileptology, Schoen Clinic Vogtareuth, Vogtareuth, Germany

**Keywords:** congenital brain injury, motor impairment, selective motor control, physical exercise, neurostimulation, neuromodulation

## Abstract

**Background:**

Impaired selective motor control, weakness and spasticity represent the key characteristics of motor disability in the context of bilateral spastic cerebral palsy. Independent walking ability is an important goal and training of the gluteal muscles can improve endurance and gait stability. Combining conventional physical excercises with a neuromodulatory, non-invasive technique like repetitive neuromuscular magnetic stimulation probably enhances effects of the treatment. This prospective study aimed to assess the clinical effects of repetitive neuromuscular magnetic stimulation in combination with a personalized functional physical training offered to children and adolescents with bilateral spastic cerebral palsy.

**Methods:**

Eight participants Gross Motor Function Classification System level II and III (10.4 ± 2y5m; 50% Gross Motor Function Classification System level II) received a personalized intervention applying functional repetitive neuromuscular magnetic stimulation (12 sessions within 3 weeks; 12,600 total stimuli during each session). At baseline and follow up the following assessments were performed: 10-m-walking-test, 6-min-walking-test, GMFM-66. Six weeks after the end of treatment the patient-reported outcome measure Gait Outcome Assessment List was completed.

**Results:**

GMFM-66 total score improved by 1.4% (*p* = 0.002), as did scoring in domain D for standing (1.9%, *p* = 0.109) and domain E for walking, jumping and running (2.6%, *p* = 0.021). Gait speed or distance walked during 6 min did not improve from baseline to follow up. Patient-reported outcome showed improvement in 4 patients in altogether 14 ratings. Caregiver-reported outcome reported benefits in 3 participants in altogether 10 ratings.

**Conclusion:**

Repetitive neuromuscular magnetic stimulation promises to be a meaningful, non-invasive treatment approach for children and adolescents with bilateral spastic cerebral palsy that could be offered in a resource-efficient manner to a broad number of patients. To further investigate the promising effects of repetitive neuromuscular magnetic stimulation and its mechanisms of action, larger-scaled, controlled trials are needed as well as comprehensive neurophysiological investigations.

## 1. Introduction

Bilateral spastic cerebral palsy (BSCP) caused by congenital or early acquired brain injury, yields a prevalence of 2.11 per 1,000 births and is one of the most common pediatric neurological disorders (1–3). Multimodal treatment aims to promote activity to foster and maintain the child’s capabilities and performance in activities of daily living supporting participation and quality of life (4–7).

Within new treatment concepts developing over the last years, the focus has changed somewhat away from *a priori* managing spasticity toward addressing the two other muscular key features of BSCP - weakness and impaired selective motor control, as well. Given the high likelihood of developing a crouch gait pattern during trajectory, training of the lower limb extensors and hip abductors becomes important to prevent and counteract the development of biomechanical malalignments of the lower extremity and pelvis, decrease compensatory trunk lean and enhance balance as well as endurance in standing and walking (8–16). In addition to orthoses that support foot leverage as well as aids supporting standing and walking, conventional and instrumented physiotherapy (e.g., robot-assisted treadmill training, whole body vibration training), are helpful approaches to improve power and endurance in children with BSCP (4).

Limitations of these treatments might occur if a child is unable to selectively control a specific muscle/muscle group, reducing its efficacy and sustainability. However, additional externally applied stimuli could favor sensorimotor processing during motor training leading to a higher efficiency to overcome these boundaries. By triggering sensorimotor network reorganization, motor learning could be fostered on the long-term. Repetitive neuromuscular magnetic stimulation (rNMS) combined with a task-specific motor training represents such a safe, well-accepted and feasible non-invasive, non-pharmacological, innovative approach of neuromodulation “from bottom up” (17–21).

rNMS is based on the principle of electromagnetic induction. A copper-winded coil is located above the target muscle/muscle groups, e.g., the gluteus muscles. The stimulation system evokes a magnetic field surrounding the stimulation coil. Based on the principle of electromagnetic induction, this magnetic fields in turn provokes an electric current in the tissue beneath (17–20). In this region, terminal motor branches are activated and a muscle contraction occurs (20). At the same time proprioceptive afferent information increases by indirect stimulation via muscle spindles and mechanoreceptors by the muscle contraction itself and directly via stimulation of terminal afferent nerve branches of the skin and joint capsule. This afferent information input results in cortico-spinal and cortico-cortico neuromodulation triggering sensorimotor network and cortical (re-) activation and (re-) organization (17–19).

Our research group developed a protocol of a functional rNMS (frNMS) training, that was applied to the gluteal muscles including children and adolescents with BSCP Gross Motor Function Classification System (GMFCS) level II and III.

This prospective study aimed to assess the clinical effects of repetitive neuromuscular magnetic stimulation in combination with a personalized functional physical training offered to children and adolescents with bilateral spastic cerebral palsy. The primary aim of the study was to assess the clinical effects of the frNMS intervention targeting to the gluteal muscle on gross motor function, in particular standing and walking capability. It was hypothesized, that gross motor function is improved after the intervention.

Here, data on the clinical effects assessed by clinician-observed, instrumented as well as a most recent patient-reported outcome measure are presented. The respective instruments were chosen to reflect the domains of activity and participation according to the international Classification of Functioning, Disability and Health, Children and Youth Version (ICF-CY) (22).

## 2. Methods

### 2.1. Ethics statement

The study protocol was approved by the institutional review board (vote 20–604). The study was conducted in accordance with the declaration of Helsinki and registered at the German Registry for Clinical Studies (DRKS00023766). Informed written consent of participants and their guardians was a perquisite for participating the study.

### 2.2. Study design

Monocenter, uncontrolled, prospective, open-label clinical study. A baseline assessment was performed within 6 days prior to the first therapy session; the clinical follow-up (FU) assessment took place within 6 days after the last session (Gross Motor Function Measure (GMFM-66), 10-meter-walking-test (10MWT), 6-min-walking-test (6MWT)) (23–27). At baseline and at 6 weeks after the last session (FU-6) participants and caregivers completed the Gait Outcome assessment List (GOAL) (28, 29).

### 2.3. Study population

Patients with BSCP, who are regularly seen in the institution’s outpatient clinic, were screened for eligibility for participating in the study. Inclusion criteria implied diagnosis of BSCP, Gross Motor Function Classification System (GMFCS) Level I to III, age between 6 and 18 years and insufficient hip extension during walking and/or standing. Exclusion criteria comprised general contraindications of magnetic stimulation (implanted biomedical devices incl. Shunt systems, ferromagnetic implants, epilepsy), confirmed attention deficit (hyperactivity) disorder, intellectual disability (IQ < 70), orthopedic surgery or injection of botulinum toxin affecting the lower extremity within the previous three months and a hipflexion contracture >15°. In case the patient was eligible to take part in the study, the patient and their caregivers were offered the functional rNMS intervention and educated about the following treatment.

### 2.4. frNMS intervention

The frNMS intervention was composed of 12 *a priori* scheduled sessions taking part within 3 weeks. All training sessions were performed by trained therapists under the supervision of a board-certified physiotherapist, who was trained in BSCP on an expert level. Every therapy session included 20 min of net stimulation time (10 min per body side). For stimulation protocol and set up of frNMS refer to Table 1 (*Grosse et al., Functional repetitive neuromuscular magnetic stimulation targeting to the gluteal muscles in children with bilateral spastic cerebral palsy – safety, feasibility, and patient-reported outcome, submitted to Frontiers in Neurology February 8th 2023*).

**Table 1:** Stimulation protocol of the frNMS intervention targeting to the gluteal muscles.

Stimulation system					
Round coil	12.5 cm diameter of the copper winding	3 Tesla max. Output	Integrated oil cooling system	Rectangular pulse shape	412 μs pulse duration
Stimulation protocol					
ON-time (train)	OFF-time (break)	Frequency	Duration	Total trains Per exc.	Total stimuli per exc.
3 s. (3 bursts per train)	6 s.	25/35 Hz alternately	2 min	14	1,155
Treatment protocol					
Set of 21 physiotherapeutic exercises focusing on the gluteal muscles (hip extension, abduction, external rotation)	5 Exercises repeated for 2 min on both body sides (including starting with “warm up”)	14 repetitions per exercise and body side	12,600 total stimuli per session during performance of physical exercises	Intensity (% of max. Output of the stimulator) was individually adopted for every exercise and body side during each session.	

cm, centimetres; Hz, hertz; max., Maximal; ms, milliseconds; Nbr., Number; sec., seconds.

### 2.5. Clinician-observed outcomes

Participants completed the 10MWT at self-selected walking speed (SSWS), followed by maximum walking speed (MWS) two times each. In the 6MWT, the distance (in meters) walked in self-selected pace for 6 min was measured and the times needed to rest (in seconds) including periods of standing or leaning against the wall were documented. The timing was stopped after having completed the six minutes or when the participant would interrupt the test because of fatigue (including sitting down). Participants were asked to wear the same footwear, orthoses or gait aid at both assessments (26, 27). In a previous study the MDCs95 (minimal detectable change 95% confidence level) for the 10MWT at maximum walking speed was reported as 4.3 s and 17.7. s for GMFCS level II and III, respectively (27). For the 6MWT MDCs95 were reported with 64 m and 47.4 m for GMFCS level II and III, respectively (27).

GMFM-66 comprises activities of five dimensions including (A) lying and rolling (4 items), (B) sitting (15 items), (C) crawling and kneeling (10 items), (D) standing (13 items) and (E) walking, running and jumping (24 items). Each item is scored on a 4-point ordinal scale ranging from 0 to 3 (0 = no task initiation; 1 = initiation but <10% of task completed; 2 = initiation and 10–99% of task completed; 3 = completion of task) (23, 24, 30, 31). Minimum clinically important differences (MCID) are available across all GMFCS levels for total score, dimension D and E (32).

### 2.6. Patient and caregiver-reported effects

At baseline and FU6, patients and their caregivers completed the Gait Outcomes Assessment List (GOAL) in its German paper-based version (28). GOAL is a questionnaire evaluating gait priorities and functional mobility of children with CP. The child and their caregivers assess the child’s performance and perception across seven domains covering altogether 48 items, respectively. Domain A: activities of daily living and independence; domain B: gait function and mobility; domain C: pain, discomfort, and fatigue; domain D: physical activities, sports and recreation; domain E: gait pattern and appearance; domain F: use of braces and mobility aids; domain G: body image and self-esteem. Results entail a total GOAL score and individual domain scores (28, 29). To this day, no MCID have been reported. For the analysis on the individual level, a score change of ≥5 points was considered as improvement based on available test–retest data (28).

### 2.7. Data management

Patient characteristics, data collected during assessments and details of treatment during frNMS sessions were documented using paper-based clinical report forms and entered electronical Microsoft Excel data sheets (Microsoft Office Professional Plus 2016, Microsoft, Redmond, Washington, USA). Data entry was cross-checked by two independent analysts.

### 2.8. Statistical analyses

The statistical analyses were conducted using Microsoft Excel (Microsoft Office Professional Plus 2016, Microsoft, Redmond, Washington, USA) and SPSS (version 26/27; IBM SPSS Statistics for Windows, Armonk, NY, USA). If participants did not perform one of the assessments, they were excluded from the respective analysis. Absolute and relative frequencies, means, standard deviations (SDs), medians, and ranges were generated for subject and intervention characteristics including patient reported and clinical outcome.

All datasets were tested for normal distribution, by using the Shapiro-Wilks test and thereafter for statistically significant changes from baseline to FU or FU-6 with adequate tests: normally distributed 10 MWT (at maximum walking speed) and 6 MWT, GMFM-66 (except dimension D), GOAL scores of caregivers (except domains A, E, and G) as well as GOAL scores of participants (except domains total, B and E) by paired *t*-test; not normally distributed 10MWT (at self-selected walking speed), GMFM-66 D, remaining GOAL scores of caregivers and participants by Wilcoxon Signed-Rank test. The level of significance was set at *p* < 0.05.

## 3. Results

Eight children and adolescents with BSCP took part in the study (5 females, mean age at baseline: 10.4 ± 2y5m; Table 2). Regarding 10MWT and 6MWT no clinically meaningful change was observed – nor on the individual, nor on the group-level (10MWT: SSWS: *p* = 0.246; MWS: *p* = 0.116; 6MWT: *p* = 0.688; Table 3).

**Table 2:** Characteristics of children with bilateral spastic cerebral palsy, who underwent the frNMS intervention.

N	Sex	Age*	GMFCS Level	Neuro-imaging	GA (w + d)	BW (g)	Previous interventions	Mobility-related treatment goals
1	M	10y 7 m	III	PVL	32 + 3	1,820	3*BoNT, 6*RAGT	To walk short distance in and outside without assistance, to climb and step down stairs by himself, perform toileting independently
2	F	8y 11 m	II	PVL	28 + 5	1,040	2*BoNT, 7*RAGT	To be able to stand upright, increased endurance for standing upright and walking without assistance, stopping from walking, turn around on the spot while walking
3	M	10y 3 m	II	MRI without corresponding finding	41 + 4	3,930	3*BoNT, 1*WBV, 7*RAGT 1*frNMS SDR in 2017	To ride a bike without training wheels, decreased body sway during walking, increased stability during walking
4	F	6y 11 m	II	PVL	29 + 3	1,140	6*BoNT, 1*RAGT	To climb and step down stairs by herself without railing, increased endurance for walking with and without orthoses, keeping left heel down when walking with orthoses, increased stability for standing when being pushed, be able to perform one-leg-jump right and left, keeping left foot on pedal while riding a bike
5	F	13y 2 m	III	PVL	29 + 1	1,355	2*BoNT, 6*RAGT	To climb stairs by herself without railing, to be able to stand upright and holding balance, increased walking endurance, increased leg stretching while walking
6	F	14y 3 m	III	PVL	26 + 2	730	1*RAGT	Increased endurance for walking, increased balance while standing and walking, walking upstairs without railing, jumping far with both legs, to be able to dress herself faster
7	F	11y 11 m	III	PVL	33 + 0	2,150	6*BoNT, 2*RAGT	Standing up with help, increased walking endurance and more effortlessly walking with support/walker/orthoses, to be able to stand free with orthoses
8	M	7y 1 m	II	PVL	40 + 0	2,975	3*BonT, 1*RAGT, 1*WBV	Increased walking endurance, increased stability while walking, walking with heels on the ground, sitting on the ground without support by her own arms, to be able to ride a bike with training wheels

BoNT, Botulinumtoxin; BW (g), birth weight (in grams); DAFO, dynamic ankle foot orthesis; F, female; GA (w + d), gestational age (in weeks and days); GMFCS, Gross Motor Function Classification System; L, left; M, male; m, months; MRI, magnetic resonance imaging; N, number of participant; PVL, periventricular leukomalacia; R, right; RAGT, Robotic Assistant Gait Training; SDR, Selective Dorsal Rhizotomy; WBV, Whole Body Vibration; y, years; *Age at baseline assessment.

**Table 3:** Clinical effects of frNMS targeting to the gluteal muscles.

Assessment		10 MWT				6 MWT		GMFM-66							
		SSWS (s)		MWS (s)		Distance (m)		D		E		Total		Percentiles	
Patient	GMFCS Level	BL	FU	BL	FU	BL	FU	BL	FU	BL	FU	BL	FU	BL	FU
1	III	---	---	---	---	---	---	84.6	84.6	36.1	**38.9**	61.5	**62.4**	85	90
2	II	8.2	9.0	4.3	4.5	393	426	82.1	82.1	70.8	**73.6**	69.6	**70.8**	55	60
3	II	8.3	7.6	3.2	3.6	500	456	89.7	89.7	86.1	87.5	77.5	78.3	75	75
4	II	11.7	9.6	4.3	4.1	324	343	82.1	82.1	77.8	77.8	72.2	72.2	85	80
5	III	12.6	10.7	8.1	5.3	350	312	79.5	**82.1**	61.1	**66.7**	67.0	**68.9**	n.a.	n.a.
6	III	9.2	8.6	7.6	7.2	392	408	79.5	79.5	65.3	**72.2**	67.7	**70**	n.a.	n.a.
7	III	25.5	14.2	14.1	12.3	170	215	43.6	**48.7**	11.1	12.5	51.6	**53.1**	40	50
8	II	9.5	10.8	3.9	2.9	618	625	84.6	**92.3**	97.2	97.2	81.9	**84.0**	>97	>97
Mean (SD)		12.1 (5.7)	10.1 (2.0)	6.5 (3.6)	5.7 (2.9)	392.4 (130.0)	397.9 (119.3)	78.2 (13.4)	**80.1 (12.5)**	63.2 (26.0)	**65.8 (25.7)**	68.6 (8.8)	**70.0 (9.4)**	--	--
		*p* = 0.246		*p* = 0.116		*p* = 0.688		*p* = 0.109		***p* = 0.021**		***p* = 0.002**		--	

Time taken for the 10-m-walking test (10MWT) for self-selected (SSWS) and maximum walking speed (MWS), distance walked in meters within the 6-min-walking test (6MWT) and total scores of GMFM-66 incl. scores of dimension D and E at baseline (BL) and follow up (FU); bold printed = change corresponding to a large [medium] sized effect of ≥ 5.3 [≥3.3]/ ≥2.4 [≥1.5] in dimension D, of ≥ 4.5 [≥2.8]/ ≥3.0 [≥1.8] in dimension E and of ≥ 1.5 [≥1.0]/ ≥1.2 [≥0.7] in total score for GMFCS level II/III, respectively. Across all GMFCS level MCID is ≥ 1.8 [≥1.2]/ ≥2.6 [≥1.6]/ ≥1.3 [≥0.8] for dimension D, E and total score, respectively (32). GMFM-66 percentiles according to (33); n.a. due to age > 12 years.

The GMFM-66 total score significantly improved from 68.6 (SD 8.8) to 70.0 (SD 9.4) (∆ + 1.4), corresponding to a large sized clinically meaningful effect (*p* = 0.002) driven by medium effect sized benefits in two and large effect sized benefits in four participants (Table 3). Referring to domains A to C, seven participants reached maximum scores (100%) at baseline without any change in performance at FU, one patient reached 98,6% at baseline and 100% at FU. In domain D, three participants experienced a clinical meaningful improvement of large effect size; regarding dimension E clinically relevant changes of medium effect size were reported in two patients, and of large effect size in two other patients (Table 3). On the group level, the score for dimension E improved from 63.2 to 65.8 (∆ + 2.6), displaying a statistically significant clinically meaningful change of large effect size (*p* = 0.021). Not any participant experienced a decrease in dimension D, E nor total score.

By GOAL, one participant reported improvement across all domains resulting in a 39% increase in his total score (Table 4). In addition, two other participants reported improvements in domain D (physical activities, sports & recreation), two participants in domain F (use of braces & mobility aids), one in B (gait function & mobility), one in C (pain, discomfort, & fatigue), and one other in G (body image and self-esteem), respectively. Caregivers reported improvements for one participant in domain A (activities of daily living & independence), B, D, E (gait pattern & appearance) and F. Further, improvement was reported for another participant in domain C, D, F and G, and another participant for domain E, respectively (Table 5). On the group level, improvements did not reach significance neither for patients nor caregivers (*p* > 0.05). A decrease of ≥5 points was reported for one patient each in domains B, D, F, and G, respectively.

**Table 4:** GOAL total and domain scores reported by participants at baseline (BL) and follow up (FU) each.

Domain		Total		A		B		C		D		E		F		G	
Patient	GMFCS Level	BL	FU	BL	FU	BL	FU	BL	FU	BL	FU	BL	FU	BL	FU	BL	FU
1	III	40.8	**56.7**^#^	43.2	**54.3**^#^	47.8	**56.7**	85.7	**100**^#^	10.4	**16.7**	30.6	**52.8**^#^	8.3	**66.7**^#^	41.7	**62.5**^#^
2	II	--	—	--	--	--	--	--	--	23.8	**54.8**^#^	--	--	75.0	**87.5**^#^	--	--
3	II	78.7	80.8	91.4	95.1	77.8	**87.0**	100	100	68.8	**54.2**^#^	66.7	66.7	83.3	**91.7**	62.5	**70.8**
4	II	**	**	**	**	**	**	**	**	**	**	**	**	**	**	**	**
5	III	74.0	74.0	93.8	93.8	80.0	80.0	97.1	97.1	47.6	47.6	58.3	58.3	--	--	62.5	62.5
6	III	59.1	59.6	87.7	85.2	61.1	61.1	69.0	**80**^#^	29.2	29.2	55.6	55.6	66.7	66.7	40.0	40.0
7	III	***	***	***	***	***	***	***	***	***	***	***	***	***	***	***	***
8	II	****	****	****	****	****	****	****	****	****	****	****	****	****	****	****	****
Mean (SD)		-	-	79.0 (24.0)	82.1 (19.0)	-	-	88.0 (14.1)	**94.3** (9.6)	36.0 (22.7)	40.5 (16.9)	-	-	58.3 (34.0)	**78.2** (13.3)	51.7 (12.5)	**59.0** (13.2)
Median		66.6	66.8	-	-	69.5	70.6	-	-	-	-	57.0	57.0	-	-	-	-
		*p* = 0.181		*p* = 0.376		*p* = 0.371		*p* = 0.187		*p* = 0.575		*p* = 1.000		*p* = 0.228		*p* = 0.235	

Bold/italic printed = increase/decrease of ≥ 5 points; # increase/decrease ≥ 10 points; participant 2 completed dimension D and F only at FU6. ** not reported as patient underwent percutaneous myofasciotomy during interval; *** participant herself was not available for long-term follow up; **** participant was not able to complete the questionnaire due to insufficient literacy in German.

**Table 5:** GOAL total and domain scores reported by caregivers at baseline (BL) and follow up (FU) each.

Domain		Total		A		B		C		D		E		F		G	
Patient	GMFCS Level	BL	FU	BL	FU	BL	FU	BL	FU	BL	FU	BL	FU	BL	FU	BL	FU
1	III	39.1	**44.1**	24.7	**40.7**^#^	37.0	**43.0**	85.7	85.7	10.4	**20.8**^#^	36.1	**41.7**	50.0	**58.3**	45.8	***29.2***^**#**^
2	II	70.5	70.9	74.1	75.3	73.0	76.0	--	--	72.2	72.2	69.4	69.4	75	*62.5*^#^	58.3	58.3
3	II	71.7	69.8	88.9	90.1	81.0	81.0	95.9	93.9	42.9	*35.4*	61.1	61.1	50.0	50.0	50.0	50.0
4	II	**	**	**	**	**	**	**	**	**	**	**	**	**	**	**	**
5	III	49.4	49.4	69.1	69.1	42.2	42.2	81.6	81.6	16.7	16.7	36.1	36.1	--	--	50.0	50.0
6	III	47.2	46.8	79.0	79.0	61.4	61.4	--	--	14.6	14.6	33.3	33.3	50.0	50.0	45.8	45.8
7	III	57.0	**64.1**	69.1	67.9	62.9	61.3	91.8	**98.0**	20.8	**31.3**^#^	58.3	58.3	58.3	**91.2**^#^	37.5	**58.3**^#^
8	II	63.3	66.6	70.4	74.1	69.0	*61.0*	95.9	98.0	40.5	38.1	30.6	**47.2**^#^	75	75	62.5	62.5
Mean (SD)		56.9 (12.3)	58.8 (11.6)	-	**-**	60.9 (16.0)	60.8 (14.7)	90.2 (6.4)	91.4 (7.4)	31.2 (22.1)	29.4 (20.6)	-	-	59.7 (12.3)	64.5 (16.0)	-	-
Median		-	-	70.4	74.1	-	-	-	-	-	-	36.1	47.2	-	**-**	50.0	50.0
		*p* = 0.170		*p* = 0.100		*p* = 0.960		*p* = 0.417		*p* = 0.504		*p* = 0.371		*p* = 0.478		*p* = 1.000	

Bold/italic printed = increase/decrease of ≥ 5 points; # increase/decrease ≥ 10 points; −, not completed; ** not reported as patient underwent percutaneous myofasciotomy during interval.

## 4. Discussion

Eight children with BSCP GMFCS level II and level III underwent a personalized frNMS intervention targeting to the gluteal muscles to improve functioning of lower limb extensors and hip abductors aiming at an improvement of balance and endurance during standing and walking (*Grosse et al., Functional repetitive neuromuscular magnetic stimulation targeting to the gluteal muscles in children with bilateral spastic cerebral palsy – safety, feasibility, and patient-reported outcome, submitted to Frontiers in Neurology February 8th 2023*).

These goals were accomplished as reflected by GMFM assessments. The increases in domain D “standing” of +1.9%, domain E “walking, running and jumping” of +2.6% and GMFM total score of +1.4% reflect clinically meaningful improvements in WHO-ICF domain activity.

As the multimodal treatment approach to BSCP already comprises a relevant number of interventions to choose from on an informed basis, it is important to set the outcomes achieved by frNMS in light to other instrumented training-based interventions and to highlight its difference against the other modalities (4).

First, robot assisted treadmill training represents an important option for children with BSCP (25, 26, 34–39). On the group level, reaching large effect sized changes in dimension D, E and GMFM-66 total score, the benefits achieved by the frNMS intervention pointed in the same direction than the effects reported for robot-assisted treadmill training timely after 12 sessions training during 3 weeks in four different publications (*n* = 14, 18, 20, and 83 children with BSCP GMFCS level I to IV, mean age (years) 8.2 ± 5.4; 11.4 ± 4.9; 11.0 ± 5.1; 10y8m ± 6y1m) (25, 26, 38, 39).

Regarding GMFCS level homogeneity, this study best compares to the report of Weinberger et al., who observed changes in GMFM in 18 children (mean age 5.9 years) affected by BSCP GMFCS level II and III (40). In their study improvement achieved during each of three treatment blocks was particular emphasized in dimension D. The participants of the current frNMS study were older and started at a relevantly higher functional level given their baseline GMFM total, dimension D and E scores than the participants of the robot-assisted treadmill training study. Interestingly, although functional levels within their specific GMFM level were already quite high at baseline, beneficial effects could still be achieved by frNMS. This may point at the importance of specifically addressing impaired or missing selective motor control as a highly relevant treatment approach. Besides counteracting weakness, the massive proprioceptive inflow caused by the externally triggered muscular contraction is highly likely promoting central network (re-) activation and reorganization. Compared to robot assisted treadmill training, frNMS is far less resource-intensive, its application is easily trained and well feasible. These advantages could make it available to a higher number of children affected by CP with a wide-spread distribution of this approach.

Secondly, whole body vibration (WBV) represents another treatment option offered in neurorehabilitation for children with CP (4, 41). By introducing side-alternating WBV to conventional physical therapy, 15 children with BSCP (mean age 9.6 years; GMFCS level not specifically reported) achieved significant improvements in dimension D and E after 12 weeks of training, with effects in dimension E being significantly higher than in the control “standard physiotherapy” group (42). Four additional WBV studies were not comparable to this frNMS study given differences in study design and set up of the intervention (continuous training over 6 months, home-based training or intensive functional blocks of interval rehabilitation) (43–46).

Neuromuscular electrical stimulation (NMES) acts very similar to repetitive neuromuscular magnetic stimulation (4). In two studies including 11 and 20 children in the experimental group (age 5 to 11 years and 8.6 ± 2.8 years, GMFCS level not specifically reported), the gluteal muscles were electrically stimulated adjuvant to conventional physical exercises over 4 to 8 weeks (47–49). In these studies, this combination for the gluteal muscles exhibited clinically meaningful benefits regarding dimension D and for dimension E (48, 49). In the study by Mohanty et al., these effects were significantly higher compared to the control group receiving conventional physical treatment only. Given the technical advantages together with the painlessness of magnetic stimulation, it is highly likely that children and therapists will favor rNMS over NMES (17–19).

Another way of non-invasive brain stimulation is the application of transcranial magnetic or direct current stimulation. Here, specifical cortical regions are targeted to facilitate or inhibit networks related to motor function and motor learning. Usually, transcranial neurostimulation is combined with physical or occupational training aiming at a promotion of mechanisms of neuroplasticity. With regard to gross motor function no studies exploring effects of transcranial magnetic stimulation (TMS) are yet available. But, for transcranial direct current stimulation two studies reported benefits in domain D and domain E when adding tDCS of the dominant or ipsilesional M1 to treadmill or virtual reality training (10 sessions a 20 min each; 10/12 children, GMFCS level II and III; mean age 8.2 ± 1.6) (50–53).

Regarding GOAL, improvement in at least one domain was reported by 4 participants and 3 caregivers, respectively. Change was >10 in 66% of participants’ and in 50% in caregivers’ improved ratings, highly probably translating to meaningful effects on the individual level for the children’s everyday lives. As the GOAL is a quite recent tool, no interpretation of the numerical change regarding a minimal clinical important difference or comparison to effects attained during other interventions are available, yet.

Almost all of the eight patients performed very well in 10MWT and 6MWT at baseline compared to the available GMFCS-appropriate reference data and to other studies reporting on children affected by BSCP (26, 27, 35, 37). Ceiling effects together with the limited sample size may have hampered discrimination of treatment effects. Further, interpretation of the walking tests is hampered by the large range of time spans the MDC95 at MWS are based on and the absence of MDC95 for SSWS (27). However, as other research groups detected significant changes in walking speed and/or endurance by these clinical outcome measures after robotic assisted/resistance treadmill training, WBV, and tDCS, larger-scaled data for frNMS should be awaited prior drawing distinct conclusions on these parameters (26, 35, 37, 41, 50, 51, 54, 55).

Different mechanisms of action are likely to promote the beneficial effects of frNMS. The combination of painless neurostimulation inducing a physiologically sized contraction together with a set of tailored exercises directly enhances power of the stimulated muscle and improves motor units’ recruitment (17, 18, 56–66). A massive proprioceptive inflow to the central nervous system is triggered - indirectly via muscle contractions through muscle spindles and mechanreceptors and directly via stimulation of terminal branches of afferent nerves (17, 18, 63–68). Centrally, sensorimotor network activation and reorganization may represent the key to promote voluntary activation of the target muscles. In addition, down regulation of spinal hyperreflexia may contribute to the positive effects of rNMS, as well (69, 70).

Two previous publications reported about a static rNMS treatment targeting the peroneal and tibial nerves in children with CP (71, 72). In the first report, 5 sessions of rNMS (1800 stimuli during each) resulted in a decrease of plantar flexor spasticity on the more affected side of five children with BSCP. Spasticity was measured by manual dynamometer assessment pre and post each session without any longstanding follow up measurements. In a succeeding report, the same protocol was applied to a boy affected by spastic hemiparesis. In this case the following observations were described: a decrease of plantar flexor spasticity, that sustained at 45 days post intervention; an increase of active and passive ankle dorsiflexion, that sustained at 15 days but not at 45 days post intervention; improved gait parameters (stride length, velocity, cycle duration, cadence), that sustained at 15 days but not at 45 days post intervention (71, 72). These observations are congruent to the improvements reported in our study regarding GMFM dimension E tasks.

In our institution we chose a very personalized frNMS approach. The definition of individual goals prior to the intervention supports self-empowered decision making and facilitates choice of the exercises. Moreover, the flexibility of the personalized set up allows for quick adaptions of training intensity and difficulty with consideration of endurance of participants during each single session.

The uncontrolled design and the limited sample size together with the personalized approach do not allow for any generalizable conclusions about the effectiveness of the frNMS intervention. All treatments were performed according to the best clinical practice by selecting exercises that contribute the most to the achievement of the goals set by the participants given their current level of functioning. However, some children may profit from longer stimulation times, a higher number of different exercises during each session, a higher stimulation intensity or more sessions within the same or a longer time frame. Regarding the choice of outcome parameters ceiling effects may have played a role to not detect any change on the group level by 10MWT and 6MWT. The timed up and go test may be a reasonable alternative for future studies, as may be the inclusion of a tool specifically designed to assess selective motor control (i.e., Selective Control Assessment of the Lower Extremity – SCALE) (73, 74). Objective diagnostic measures to assess for clinical and neurophysiological effects (e.g., instrumented posturography, 3D gait analysis, EMG monitoring, muscle ultrasound, TMS mapping, fMRI) should be implemented within a future randomized, controlled trial. Future investigations on biomarkers serving as biological predictors of response as well as reflectors of treatment responsiveness are highly needed to stratify therapeutic offers and resources in the most effective and efficient way (39, 52). Although based on a limited sample size, the current findings are a first step to operationalize endpoints and to calculate sample size based on effects sizes for large-scale, controlled clinical trials to further assess the effectiveness of the frNMS intervention.

## 5. Conclusion

For children affected by BSCP, motor training is often hampered by impaired selective motor control of the target muscles. The combination of physical exercise and repetitive neuromuscular magnetic stimulation (rNMS) bypasses this challenge. This prospective pilot study aimed to assess clinical effects of a newly developed functional rNMS intervention targeting to the gluteal muscles with regard to improve gross motor function, in particular standing and walking capability. The externally induced muscular contraction provokes a massive proprioceptive information inflow to the central nervous system and promoted motor achievements of clinically meaningfulness in this small sampled study. Compared to other technical supported training methods, rNMS could be easily provided to a broad number of paediatric patients as its application is not limited to tertiary centers. frNMS as developed by our research group has the potential to become an important treatment approach in the armentarium of comprehensive motor rehabilitation programs for children and adolescents affected by congenital or acquired brain injury.

## Data availability statement

The raw data supporting the conclusions of this article will be made available by the authors, without undue reservation.

## Ethics statement

The studies involving human participants were reviewed and approved by Medical Faculty of LMU; vote 20-604. Written informed consent to participate in this study was provided by the participants’ legal guardian/next of kin.

## Author contributions

MB, FH, SS, SB, MH, and LG were performed the conceptualization. JS, MS, AM, BP, UB, BW, CB, MB, and LG did the methodology and data collection. JS, MS, and CB did the data curation. JS, MS, CB, LG, MB, FH, AS, SS, SB, and MH did the formal analysis and interpreted the data. JS, MS, LG, and MB wrote and drafted the original manuscript. All authors wrote, reviewed and edited the final manuscript. JS, MS, and CB were provided the visualization. MB, FH, SB, and SS were performed the supervision. CB and MB administrated the project. MB did the funding acquisition. All authors contributed to the article and approved the submitted version.

## Funding

This publication did not receive any specific grant from funding agencies in the public, commercial, or not-for-profit sectors. MB’s research concerning neuromodulation in congenital and acquired brain injury was supported by the Stiftung für Natur und Kinder, Germany and the Foundation of the Medical Faculty of the Ludwig-Maximilians University, Munich, Germany.

## Conflict of interest

LMU Center for Children with Medical Complexity, Munich Germany is provided by an emFieldPro magnetic stimulator by Zimmer MedizinSysteme GmbH (Neu-Ulm, Germany). FH has received speaker’s honoraria from Allergan PLC, Desitin, Ipsen Biopharmaceuticals, Merz Therapeutics, and Novartis and unrestricted educational grants from Allergan and Merz Therapeutics. SB has received consultant fees from Ipsen Pharma and Merz Therapeutics and speaker fees from Ipsen Pharma, Pharm Allergan, and Merz Therapeutics. SS has received speaker’s honoraria from and participated in advisory boards for Allergan PLC, Ipsen Biopharmaceuticals, and Merz Therapeutics. MB’s research on neuromodulation is supported by research grants from the HABA Foundation, the Deutsche Rentenversicherung, the Deutsche Migräne und Kopfschmerzgesellschaft, CSL Behring, and the ZNS-Hannelore Kohl Foundation and a research scholarship of the Bavarian Gender Equality Grant of the Free State of Bavaria, Germany.

The remaining authors declare that the research was conducted in the absence of any commercial or financial relationships that could be construed as a potential conflict of interest.

## Publisher’s note

All claims expressed in this article are solely those of the authors and do not necessarily represent those of their affiliated organizations, or those of the publisher, the editors and the reviewers. Any product that may be evaluated in this article, or claim that may be made by its manufacturer, is not guaranteed or endorsed by the publisher.

## Figures and Tables

**Table:** 

6MWT	6-min-walking-test
BSCP	Bilateral spastic cerebral palsy
CP	Cerebral palsy
domain A	Activities of daily living & independence
domain B	Gait function & mobility
domain C	Pain, discomfort, & fatigue
domain D	Physical activities, sports & recreation
domain E	Gait pattern & appearance
domain F	Use of braces & mobility aids
domain G	Body image and self-esteem
frNMS	Functional repetitive neuromuscular magnetic stimulation
FU	Follow-up
FU-6	6 weeks after the last session
GMFCS	Gross Motor Function Classification System
GMFM-66	Gross Motor Function Measure
GOAL	Gait Outcome assessment List
ICF-CY	International Classification of Functioning, Disability and Health, Children and Youth Version
MCID	Minimum clinically important differences
MDCs95	Minimal detectable change 95% confidence level
NMES	Neuromuscular electrical stimulation
rNMS	Repetitive neuromuscular magnetic stimulation
SCALE	Selective Control Assessment of the Lower Extremity
SDs	Standard deviations
SSWS	Self-selected walking speed
TMS	Transcranial magnetic stimulation
WBV	Whole body vibration
